# Antibody Therapies in Autoimmune Encephalitis

**DOI:** 10.1007/s13311-021-01178-4

**Published:** 2022-01-20

**Authors:** I. Smets, M. J. Titulaer

**Affiliations:** Erasmus University Medical Center, Rotterdam, The Netherlands

## Abstract

Autoimmune encephalitis (AE) comprises a heterogeneous group of disorders in which the host immune system targets self-antigens expressed in the central nervous system. The most conspicuous example is an anti-*N*-methyl-d-aspartate receptor encephalitis linked to a complex neuropsychiatric syndrome. Current treatment of AE is based on immunotherapy and has been established according to clinical experience and along the concept of a B cell-mediated pathology induced by highly specific antibodies to neuronal surface antigens. In general, immunotherapy for AE follows an escalating approach. When first-line therapy with steroids, immunoglobulins, and/or plasma exchange fails, one converts to second-line immunotherapy. Alkylating agents could be the first choice in this stage. However, due to their side effect profile, most clinicians give preference to monoclonal antibodies (mAbs) directed at B cells such as rituximab. Newer mAbs might be added as a third-line therapy in the future, or be given even earlier if shown effective. In this chapter, we will discuss mAbs targeting B cells (rituximab, ocrelizumab, inebulizumab, daratumumab), IL-6 (tocilizumab, satralizumab), the neonatal Fc receptor (FCRn) (efgartigimod, rozanolixizumab), and the complement cascade (eculizumab).

**Supplementary Information:**

The online version contains supplementary material available at 10.1007/s13311-021-01178-4.

## Introduction

Autoimmune encephalitis (AE) comprises a heterogeneous group of disorders in which the host immune system targets self-antigens expressed in the central nervous system (CNS). The antibody epitopes can be located intracellularly which is associated with the presence of a concurrent tumor (Chapter 8) or extracellularly on which we will focus in this chapter. In 2007, anti-*N*-methyl-D-aspartate receptor (NMDAR) antibodies were the first to be discovered and linked to a complex neuropsychiatric syndrome previously not thought to be immune-mediated [1]. Meanwhile, more and more autoantibodies directed at neuronal cell surface antigens have been identified and associated with specific neurological phenotypes [2]. Anti-leucine-rich glioma-inactivated protein 1 (LGI1) encephalitis typically manifests with subtle faciobrachial dystonic or other focal seizures followed by memory disturbances [3–6]. Anti-contactin-associated protein-like 2 (CASPR2) encephalitis is characterized by a protracted course, and anti-alpha-amino-3-hydroxy-5-methyl-4-isoxazolepropionic acid receptor (AMPAR) antibodies induce an often paraneoplastic, treatment-responsive limbic encephalitis which frequently relapses [6–8]. The presence of tumor varies per type of antibody. Some antibodies are commonly associated with tumors whereas in other antibody-associated syndromes, tumors are rare or absent [9].

The circulating neuronal cell surface antibodies are aimed at extracellular proteins which have a direct pathogenic effect by influencing the antigen function or causing antigen internalization in the absence of cell destruction [10–13]. By counteracting the autoimmune response with immunosuppressive drugs, most patients, even those who are severely ill, make a substantial improvement [1, 5, 14, 15]. This is different from AE associated with antibodies targeting intracellular epitopes (i.e., paraneoplastic syndromes) in which the effect of immunotherapy is less established.

Current treatment of AE is based on immunotherapy and has been established according to clinical experience and along the concept of a B cell-mediated pathology induced by highly specific antibodies to neuronal surface antigens [16–18]. The available evidence to treat AE is mainly based on retrospective data, and (randomized) clinical trials are largely lacking. This is due to the relative novelty of AE as well as the rarity of these diseases making large-scale international collaborations to study treatment effects necessary. Currently, we have most evidence-based experience treating anti-NMDAR encephalitis following the publication of one large, partially prospective cohort study [15]. For the other AE types, mainly smaller cohorts or cases series have been published [4, 5, 8, 19, 20].

In general, immunotherapy for AE follows an escalating approach [16–18]. In the acute phase, first-line immunotherapy is initiated which mostly involves a combination of high-dose steroids and immunoglobulins or plasma exchange. When first-line therapy fails, one converts to second-line immunotherapy. Alkylating agents such as cyclophosphamide could be the first choice in this stage. However, due to their side effect profile, most clinicians give preference to monoclonal antibodies (mAbs) directed at B cells such as rituximab [21]. Newer mAbs might be added as a third-line therapy in the future, or be given even earlier if shown effective.

In this chapter, we will discuss mAbs targeting B cells (rituximab, ocrelizumab, inebulizumab, daratumumab), IL-6 (tocilizumab, satralizumab), the neonatal Fc receptor (FCRn) (efgartigimod, rozanolixizumab), and the complement cascade (eculizumab) (Table 1).

**Table 1:** Monoclonal antibodies used or under investigation for the treatment of autoimmune encephalitis (*RCT* randomized controlled trial, *AE* autoimmune encephalitis, *anti-NMDAR* anti-*N*-methyl-D-aspartate receptor, *anti-LGI1* anti-leucine-rich glioma-inactivated protein 1, *anti-CASPR2* anti-contactin-associated protein-like 2, *anti-GAD65* anti-glutamic acid decarboxylase 65)

	**AE subtype**	**Evidence**
**B cells**		
Rituximab	Anti-NMDAR	Prospective multi-center cohort study [15], observational cohort [22], meta-analysis [23]
	Anti-LGI1, anti-CASPR2, anti-GAD65	Observational cohorts [8, 20, 22, 24]
Ocrelizumab	AE (no antibody specified)	Ongoing RCT (NCT03835728)
Inebulizumab	Anti-NMDAR	Ongoing RCT (NCT04372615)
Daratumumab	Anti-NMDAR Anti-CASPR2	Case report [25] Case report [26]
**IL-6**		
Tocilizumab	Anti-CASPR2 AE (no antibody specified)	Case report [27] Observational cohort [28], prospective single-center cohort study [29]
Satralizumab	–	Phase 2b trial pending
**Neonatal Fc receptor**		
Rozanolixizumab	Anti-LGI1	Ongoing RCT (NCT04875975)
Efgartigimod	–	–
**Complement cascade**		
Eculizumab	–	–

## Monoclonal Antibodies Targeting at B cells

### Rituximab in Anti-NMDAR Encephalitis

#### Mode of Action in Anti-NMDAR Encephalitis

Rituximab is a chimeric mAb targeting the CD20 surface antigen, a glycoprotein primarily found on the surface of B cells. It reduces both naïve and memory B cells through antibody-mediated cellular toxicity, complement activation, and induction of apoptosis [30]. Rituximab also removes antibody-producing plasma cells and consequentially lowers circulating anti-NMDAR antibody levels. As rituximab does not target long-lived mature plasma cells in the CNS, there will be prolonged presence of antibodies albeit at lower levels [30–32]. The long-term effects of the drug are most likely mediated by the deletion of the antigen-specific memory B cell populations and prevention of the formation of new plasmablasts which secrete the pathogenic antibodies [30]. Although subject to individual variation, peripheral blood B cell counts begin to increase from week 24, and evidence for repopulation is observed in the majority of patients by week 40 [33, 34]. Common rituximab dosing regimens include 375 mg/m^2^ weekly for 4 weeks or two doses of 1000 mg administered 2 weeks apart [16].

#### Timing

In patients without clinical improvement or with only minimal improvement 2 weeks after the start of first-line treatment, it is recommended to proceed with second-line therapies [16, 18]. We acknowledge this is different from the 4-week period reported in the methods of the only partially prospective, partially retrospective cohort study done in anti-NMDAR encephalitis [15]. However, in our experience, patients will not show marked improvements between week 2 and 4 after start of a first-line therapy, if improvement did not already start by day 14 [18]. This is also suggested by the results of a meta-analysis of mostly case reports and small case series in children with anti-NMDAR encephalitis. The median time from symptom onset to initiation of treatment was 15 vs. 21 days in children who recovered completely compared to children with remaining deficits at follow-up, respectively [35]. The amount of intrathecal antibody synthesis might account for this lack of response to first-line immunotherapy by a large part of anti-NMDAR encephalitis patients [32]. In patients with an anti-NMDAR encephalitis relapse, we recommend to treat with a combination of first- and second-line treatment independent of the response to first-line therapies alone [17, 18]. Although it is an option to dismiss second-line therapy and to initiate azathioprine or mycophenolate to prevent future relapses, rituximab is preferred given the faster onset of action [17]. Also, rituximab is associated with lower risk of relapses after treatment [15], while these data are lacking for azathioprine or mycophenolate. For other types of AE, this is less clear, although long-term use with azathioprine or mycophenolate seems to be associated with lower relapse risks [4].

#### Effectiveness

There are no studies comparing first-line therapies with upfront use of rituximab. The current escalation approach is based on a study of 472 patients with anti-NMDAR encephalitis treated with immunotherapy. Among the 221/472 (47%) who did not improve at 4 weeks of initiation of first-line therapies, the 125 individuals who received second-line therapies had significantly better improvement compared to the 96 individuals who did not: at 12 months follow-up 61% vs. 44% had improved to mRS 0–2, and at 24 months 78% vs. 55%, respectively [15]. In this study, the choice of adding second-line immunotherapy was not based on standardized criteria. Nonetheless, no differences in severity of the initial symptoms, duration of ICU stay, age, gender, ethnicity, and delay of initial immunotherapy were seen between patients who failed on first-line therapy versus those who did not [15]. Recently, a meta-analysis of 14 retrospective and prospective case series summarizing 277 patients with AE (89% anti-NMDAR+) concluded that rituximab had a good effect on functional outcome and prevention of relapses [23]. In a retrospective AE cohort, anti-NMDAR patients treated with rituximab (*n* = 81) were affected more severely at baseline but had reached independent living more frequently compared to untreated patients (*n* = 61) (94 vs. 88%, respectively) [22]. About 10% of the patients are refractory to a combination of first- and second-line therapy [15, 36].

#### Duration of Treatment

The relapse risk in anti-NMDAR encephalitis is estimated to be 12% within 2 years, and the large majority of the relapses will be milder than previous episodes [15]. Two other retrospective cohorts showed relapse rates of 16% and 17% after a median follow-up of 30 and 24 months, respectively [22, 37]. The chance of relapses after second-line immunotherapy, including rituximab, is however much smaller [15]. Because of these observations, there is currently no rationale to repeat rituximab at regular intervals after induction therapy [17, 18]. An exception can be made in the rare patients with relapses despite second-line immunotherapy. If tumor screening comes back negative, repetition of rituximab could be considered to avoid new relapses [38]. This could be an neuromyelitis optica spectrum disorder (NMOSD) scheme (repeated every 6 months or based on B cell repopulation) or an individual repetition scheme based on the time between onset and relapse.

#### Side Effects

In a retrospective study of 161 patients with AE who received rituximab, adverse events of rituximab were infusion-related reactions in 6.7% and infections, all pneumonia, in 11.3% [39]. No life-threatening or recurrent infectious occurred [39]. During the recent SARS-CoV-2 pandemic, the reduced vaccination response after treatment with rituximab and other anti-CD20 mAb was highlighted, although most of these patients received prolonged treatments for years [40, 41]. As the majority of people with anti-NMDAR encephalitis do not need repeated rituximab cycles, it is unclear whether these data will be similar in AE patients. Options are to postpone vaccination until B cell repopulation has occurred [40] or provide vaccines when available and check for SARS-CoV2 antibodies to monitor vaccine response.

#### Treatment in in Patients Younger than 18 Years

Treatment in children is comparable to adults, as a similar escalation approach is being used [18, 42]. Results of immunotherapy in children are slightly better than in adults. This might be because treating physicians are inclined to treat earlier and more aggressively [15, 43], but also because recovery can be better due to better plasticity [15, 37]. Rituximab is generally well tolerated, although infusion reactions affect about 12% of children, and serious infectious side effects do occasionally occur [44]. Of 144 children given rituximab for inflammatory CNS indications, 4 patients had grade 4 (disabling) or 5 (death) infectious adverse events [44]. Importantly, dosing schedules for rituximab are less well studied in children, and B cell repopulation might be faster in younger children [44, 45].

#### Intrathecal Administration

Inaccessibility of the inflammation compartmentalized to the CNS may underlie the lack of efficacy of immunomodulatory treatments in AE [13, 46]. This has prompted clinicians to administer drugs intrathecally. However, from studies in multiple sclerosis (MS), we learned that intrathecal rituximab has a profound effect on peripheral B cell numbers without consistent effect on cerebrospinal fluid B cell counts and markers [47–49]. Moreover, there was no effect of intrathecal rituximab on the presence of leptomeningeal enhancement in progressive MS patients [50]. Presumably, there are insufficient complement and effector cells for antibody-dependent cellular cytotoxicity in the intrathecal compartment [51]. There is one case report of an anti-NMDAR encephalitis resistant to intravenous rituximab at day 32 post-infusion who started to improve days after intrathecal administration of the same drug [52]. However, a delayed beneficial effect of intravenous rituximab and steroids administered previously is likely as it is known that improvement after intravenous rituximab can take a while [37].

### Rituximab in other Autoimmune Encephalitis

The therapeutic schedules for other types of AE are based on mainly retrospective cohort studies and expert opinions [16–18, 42]. Because the disease course is in most types less fulminant and relapse rates might be different, these deviate somewhat from the treatment of anti-NMDAR encephalitis. In case of no or inadequate treatment response, switching to another type of first-line treatment or proceeding to second-line treatment such as rituximab or cyclophosphamide is advised. The choice to escalate and the timing of this escalation depend on the disease severity, symptoms to improve, and the antibody involved. In a retrospective German cohort, 26 out of 61 (43%) patients with anti-LGI1, 11 out of 25 (44%) with anti-CASPR2, 31 out of 84 (37%) with anti-glutamic acid decarboxylase 65 (GAD65), and 81 out of 142 (57%) with anti-NMDAR encephalitis were treated with rituximab [22]. Observational single-center cohorts reported lower rates of rituximab use: 5/28 patients with anti-CASPR2 [8], 3/28 patients with anti-GAD65 [24], and 3/14 patients with anti-LGI1 encephalitis [20]. Although no standardized measurement of effectivity has been done, patients with anti-LGI1 and anti-CASPR2 seem to respond well to rituximab [22]. Furthermore, superiority of first- and second-line immune therapies over anti-epileptic treatment to control seizures in AE has clearly been established [4, 53]. The effectivity of rituximab in anti-GAD65 encephalitis is less established [22]. This is in line with the overall lesser response rate to immunotherapy for this disorder compared to encephalitis caused by antibodies targeting extracellular neuronal structures [24].

### Ocrelizumab in Autoimmune Encephalitis

Ocrelizumab is a humanized mAb targeting the large loop of the CD20 molecule which is partially overlapping with the epitope targeted by rituximab [54]. Compared to rituximab, ocrelizumab has less complement-dependent cytotoxicity and more antibody-dependent cellular cytotoxic activity [51]. The latter might explain the longer times needed for median B cell repopulation (72 weeks) with ocrelizumab vs. rituximab (40 weeks) [33, 55]. The clinical significance of these differences is unknown. There is currently an ongoing placebo-controlled trial to establish ocrelizumab’s efficacy in subjects with a new diagnosis of AE (NCT03835728).

### Inebilizumab in Autoimmune Encephalitis

Inebilizumab is a humanized IgG1 mAb against the CD19 B cell surface antigen. Compared to rituximab, inebilizumab does not only deplete CD20^+^ B cells but also CD20^−^ plasmablasts and plasma cells which results in more extensive and sustained suppression of B cells [56]. A recently finished phase 2/3 study for treating NMOSD showed to be very effective. The latter is another autoimmune disease characterized by the presence of anti-aquaporin-4 (AQP4) antibodies similar to anti-NMDAR antibodies that target a surface protein [56]. Of 174 participants in the inebilizumab treatment arm, 21/174 (12%) relapsed over the course of the trial compared with 22/56 (39%) in the control arm (HR 0.27; 95% CI 0.15–0.50) [57]. In moderate to severe anti-NMDAR encephalitis, the ExTINGUISH Trial will randomize 116 participants to receive either inebilizumab or placebo in addition to first-line therapies (NCT04372615).

### Daratumumab in Autoimmune Encephalitis

Daratumumab is an anti-CD38 monoclonal therapeutic antibody approved for treatment of refractory multiple myeloma and acting by depletion of plasma cells and modification of various T cell functions. Daratumumab has been applied in a case of anti-CASPR2 encephalitis that remained unresponsive to standard immunotherapies [26]. After initial clinical improvement and reduction of systemic and intrathecal antibody titers, the patient died due to septicemia. A second case report in anti-NMDAR encephalitis showed clinical improvement 2 months after initiation of daratumumab and after previous unsuccessful treatment with glucocorticoids, plasma exchange, intravenous immunoglobulins, rituximab, and bortezomib [25]. Given the sequential immunosuppressive treatment in this case, it is difficult to ascribe the clinical improvement to daratumumab alone. Remarkably, daratumumab had only minimal effect on serum anti-NMDAR titers despite falling IgG levels which incites questions about the mode of action of the drug in AE. Currently, the data are insufficient to provide proper recommendations.

## Monoclonal Antibodies Targeting IL-6

### Tocilizumab in Autoimmune Encephalitis

Tocilizumab is a humanized mAb targeting both soluble- and membrane-bound interleukin-6 receptor (IL-6R), and consequently repressing the B cell proliferation and differentiation into antibody-producing cells [58, 59]. IL-6 facilitates also other inflammatory cascades involving cytotoxic, helper, and regulatory T cells, which all contribute to autoimmunity and increase blood–brain permeability [58, 59]. Tocilizumab is an established treatment in rheumatoid arthritis and Castleman’s disease [59] and was effective in NMOSD. In the NMOSD phase 2 trial, median time to first relapse was significantly longer with tocilizumab than with azathioprine (79 vs. 57 weeks). At 60 weeks, risk for relapse was significantly lower with tocilizumab (HR 0.27; 95%CI 0.12–0.61) [60]. Tocilizumab also showed its efficacy in a case series of 8 individuals with NMOSD resistant to B cell depletion [61].

In AE, the evidence for tocilizumab is still emerging. A case of anti-CASPR2 encephalitis was successfully treated with tocilizumab as a first second-line therapy instead of B cell depletion [27]. In people with clinically suspected AE, a retrospective study showed that patients who received tocilizumab (*n* = 10) had better outcomes at 24 months than individuals who continued on rituximab (*n* = 10) or who received no further immunotherapy (*n* = 6) [28]. A single-center cohort study from the same group observed better 3-month and 1-year outcomes in anti-NMDAR encephalitis patients (*n* = 78) treated with tumor removal, steroids, immunoglobulins, rituximab, and tocilizumab (T-SIRT) within 1 month of onset compared to other regimens within the same time period [29]. Caveats suggesting caution in the interpretation of both studies are the chosen statistical analyses, small patient numbers, selection bias in patient assignment to the study groups, and confounding by the short clinical follow-up after rituximab administration in patients who also received tocilizumab. In addition, there were relatively high rates of infectious complications in people receiving the T-SIRT regimen (pneumonia 66.7%, neutropenia 21.2%) which is an unnecessary hazard in people that would have responded well to T-SIR. Confirmation by further studies in larger samples with more uniformity in AE diagnosis and timing of tocilizumab is necessary. Of note, tocilizumab increases the risk of infection, and it hampers the recognition of an infection by diminishing the fever response and the levels of C-reactive protein [62]. For this reason, clinicians must have increased awareness for systemic infection in treated patients, especially in those treated with multiple immunomodulating drugs.

### Satralizumab in Autoimmune Encephalitis

Satralizumab is a subcutaneously administered humanized mAb that binds to both membrane-bound and soluble IL-6R and prevents the binding of IL-6. This results in blocking of the IL-6-signaling pathways that are involved in inflammation. It was designed on the basis of tocilizumab with a novel antibody-recycling technology, allowing for increased duration of antibody circulation [56]. In the SAkuraSky stuy, investigating NMOSD patients, of sartralizumab plus background immunotherapy, 8/41 (20%) participants in the treatment arm relapsed over the course of the trial compared with 18/42 (43%) controls (HR 0.38; 95% CI 0.16–0.88) [63]. In the SAkuraStar study, 19/63 (30%) participants in the treatment arm relapsed over the course of the trial compared with 16/32 (50%) controls (HR 0.45; 95% CI 0.23–0.89) [64]. No data exist in AE, but a phase 2b randomized clinical trial is pending.

## Monoclonal Antibodies Targeting the Neonatal Fc Receptor

Efgartigimod is a human IgG1 antibody Fc-fragment that has increased affinity to FcRn compared with endogenous IgG while retaining the characteristic pH dependence. It outcompetes endogenous IgG binding, thereby reducing IgG recycling and increasing IgG degradation [65, 66]. Rozanolixizumab, a subcutaneously infused mAb that specifically targets FcRn, prevents IgG recycling by inhibiting the interaction of FcRn with IgG and leads to unbound IgG being eliminated [67]. The mode of action of both drugs is thus similar to immunoglobulins and plasma exchange but with the advantage of a reduced treatment burden which allows them to be used as both acute rescue and maintenance treatment. These drugs have been effective in phase 2/3 trials of patients with myasthenia gravis, another IgG antibody–mediated disease [65, 66, 68]. A phase 3 trial with rozanolixizumab is currently recruiting [68]. Phase 2 clinical trials are ongoing in patients with anti-LGI1 encephalitis (NCT04875975) and myelin oligodendrocyte glycoprotein (MOG) antibody-associated disease (NCT05063162).

## Monoclonal Antibodies Targeting the Complement Cascade

Eculizumab is a humanized mAb that inhibits the terminal complement protein C5 and prevents its cleavage into C5a, which is proinflammatory, and C5b, which coordinates the formation of membrane attack complex. There is already wider experience with eculizumab in treating myasthenia gravis [69, 70], and eculizumab was highly effective in NMOSD. In the phase 3 trial, 3/96 (3%) NMOSD patients relapsed over the course of the trial compared with 20/47 (43%) in the control arm (HR 0.06; 95% CI 0.02–0.20) [71]. Main safety concerns include risk of infections, particularly with encapsulated bacteria [71]. Eculizumab may be an option for treatment of subtypes of AE, although the evidence for complement-mediated neuronal toxicity occurring in AE is not substantial. There is some evidence from a post-mortem study for complement-mediated neuronal toxicity in anti-CASPR2 and anti-LGI1 encephalitis. However, this probably does not apply to anti-NMDAR encephalitis [32, 72, 73].

## Conclusion and Future Directions

Antibodies targeting the CD20 epitope on the surface of B cells are the cornerstone of second-line treatment in AE, originally combined with cyclophosphamide. They have a favorable side effect profile compared to second-line treatments with a different mode of action. Moreover, rituximab has shown to be effective in anti-NMDAR encephalitis as well as in many other AE cases when first-line therapies fail. However, anti-CD20 mAb do not deplete long-lived plasma cells which might underlie treatment-refractory cases and the occurrence of (early) relapses. Therefore, there is an emerging repertoire of mAb that aim to directly (inebulizumab, daratumumab) or indirectly (tocilizumab, sartralizumab) target long-lasting plasmablasts or plasma cells with or without additional B cell depletion. Alternatively, the plasma cell products (i.e. pathogenic antibodies) can be removed with mAb targeting the FcRn (efgartigimod, rozanolixizumab). mAbs that disrupt the complement cascade (eculizumab) are also explored but have fewer biological underpinnings. Future trials are needed to understand whether these third-line treatments are effective, when they need to be initiated and whether they have an acceptable safety profile in combination with previous rituximab administration.

## Supplementary Information

Below is the link to the electronic supplementary material.

Supplementary file1 (PDF 499 KB)
